# Engineering Chimeric Antigen Receptor T Cells against Immune Checkpoint Inhibitors PD-1/PD-L1 for Treating Pancreatic Cancer

**DOI:** 10.1016/j.omto.2020.05.009

**Published:** 2020-05-26

**Authors:** Ching-Yao Yang, Ming Huei Fan, Carol H. Miao, Yi Jen Liao, Ray-Hwang Yuan, Chao Lien Liu

**Affiliations:** 1 Department of Surgery, National Taiwan University Hospital, and College of Medicine, National Taiwan University, Taipei 100, Taiwan; 2 School of Medical Laboratory Science and Biotechnology, College of Medical Science and Technology, Taipei Medical University, Taipei 11031, Taiwan; 3 PhD Program in Medical Biotechnology, College of Medical Science and Technology, Taipei Medical University, Taipei 11031, Taiwan; 4 Center for Immunity and Immunotherapies, Seattle Children’s Research Institute, Seattle, WA 98101, USA; 5 Department of Surgery, National Taiwan University Hospital Biomedical Park Hospital, Hsinchu County 30261, Taiwan

**Keywords:** PD-1, PD-L1, ACR, CAR, PDAC

## Abstract

Pancreatic ductal adenocarcinoma (PDAC) is an aggressive disease with a 5-year survival rate of 9%. Major obstacles to successful treatment of pancreatic cancer are the immunosuppressive tumor microenvironment (TME) and antigenic complexity or heterogeneity. Programmed death-ligand 1 (PD-L1) is expressed on PDAC and immunosuppressed cells within the TME, providing suitable immunotherapy targets. We applied a chimeric antigen receptor (CAR) strategy to target immune checkpoint programmed death-1 (PD-1)/PD-L1 interactions. Lentiviral vectors were used to express the extracellular domain of human PD-1 (PD-1-CD28-4-1BB activating chimeric receptor [PD1ACR]) or the single-chain variable fragment (scFv) region of anti-PD-L1 (PDL1CAR) that binds to PD-L1, and each was fused to intracellular signaling domains containing CD3 zeta, CD28, and 4-1BB (CD137). Both engineered CAR T cells recognized and eliminated PD-L1-overexpressing CFPAC1 cells efficiently at approximately 80% *in vitro*. Adoptive transfer of both CAR T cells enhanced T cell persistence and induced specific regression of established CFPAC1 cancer by >80% in both xenograft and orthotopic models. Ki67 expression in tumors decreased, whereas proinflammatory cytokines/chemokines increased in CAR T cell-treated mouse sera. PD1ACR and PDL1CAR obtained a similar therapeutic efficacy. Thus, these armed third-generation PD-L1-targeted CAR T cells confer antitumor activity and the ability to combat T cell exhaustion, providing a potentially new and innovative CAR T cell immunotherapy against pancreatic cancers.

## Graphical Abstract

**Figure:**
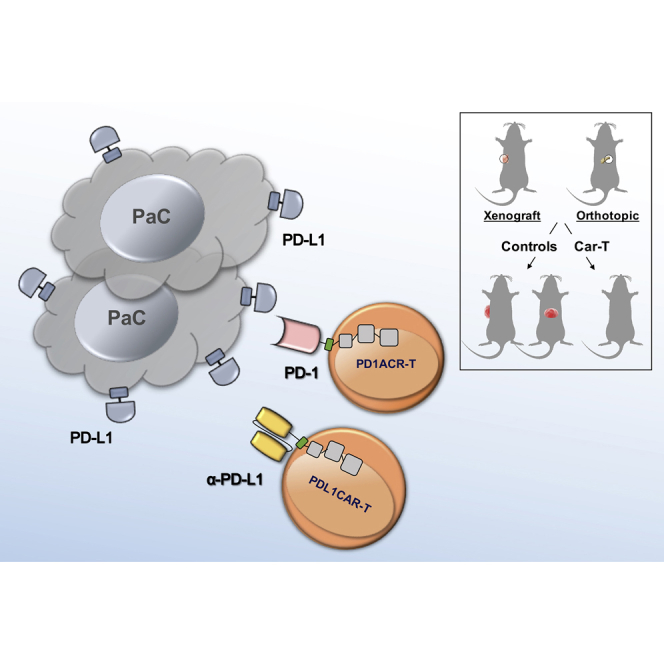

## Teaser

Yang et al. developed armed third-generation PD-L1-targeted ACR/CAR T cells utilizing a PD-1/PD-L1 immune checkpoint axis. Both engineered CAR T cells significantly improved tumor control and survival in models of PD-L1-expressed pancreatic cancers.

## Introduction

Pancreatic ductal adenocarcinoma (PDAC) remains one of the most aggressive and intractable human malignant tumors and a leading cause of cancer-related deaths (estimated 7.3% in 2018) worldwide.^1^^,^^2^ Patients with PDAC have a median survival rate of 5 months after diagnosis, and the overall 5-year survival rate is <9%.^2^^,^^3^ The overall mortality rate has not shown any significant improvement since the early 1970s, when conventional therapeutic approaches, including surgical resection, radiotherapy, chemotherapy, and a combination of these treatments, were used.^4^ PDAC is highly resistant to therapeutics because of the low burden of mutation and the presence of multiple immunosuppressive cells infiltrated in the tumor microenvironment (TME),^5^, ^6^, ^7^ considered as an “immune-quiescent” neoplasm. Therefore, cancer immunotherapy is one of the biggest breakthroughs in PDAC treatment.^8^

The programmed death-1 (PD-1)/programmed death-1 ligand-1 (PD-L1) axis is a well-known immune checkpoint inhibitor pathway. PD-1 is a T cell coinhibitory receptor, which is expressed on a large proportion of tumor-infiltrating lymphocytes in many different tumor types;^9^ its major ligand PD-L1 (also known as B7-H1 and CD274)^10^^,^^11^ is selectively expressed on many tumors,^12^^,^^13^ including PDAC, and on cells within the TME, such as macrophages and the stromal cell subset, in response to inflammatory stimuli.^14^^,^^15^ Normally, the PD-1/PD-L1 pathway serves as a negative feedback mechanism to control the immune system following an inflammatory response. Binding of PD-1 and PD-L1 initiates T cell anergy or death (so-called T cell exhaustion), thereby reducing the presence of activated effector T cells,^16^ whereas blockade of the interaction between PD-1 and PD-L1 reverses effector T cell exhaustion, thereby reinforcing antitumor activities.^17^^,^^18^ Currently, immunotherapy is an essential treatment for many cancer types, and recent preclinical and clinical evidence^19^, ^20^, ^21^ has shown promise in treating cancer by utilizing checkpoint inhibitors, including anti (α)-PD1, αPD-L1, and αCTLA4 antibodies, and vaccines. However, the clinical trial for PDAC achieved only very limited success because of weak, naturally occurring antitumor T cell immune responses.^22^ The induction of productive tumor-specific T cell immunity is a multiple-step process, and it is particularly challenging to establish a robust antitumor response in solid tumor due to its immunosuppressive TME.

Adoptive cellular therapies involving the infusion of effector immune cells into patients have generated remarkable responses in some cancers,^23^ and chimeric antigen receptor (CAR) T cell therapy represents a promising therapeutic modality for some difficult cancers, including PDAC. CARs are linked to an extracellular ligand recognition domain, typically a single-chain variable fragment (scFv), conjugated to intracellular signaling domains containing CD3ζ chain and one or more costimulatory domains, such as CD28 and 4-1BB (CD137 or TNFRS9).^24^^,^^25^ CAR scFv on T cells confers the ability to directly recognize cancer antigens in a major histocompatibility complex (MHC)-restricted manner, and CAR-specific recognition/binding to tumor antigen drives CAR T cell activation and tumor cell killing.^26^

**Figure 1:**
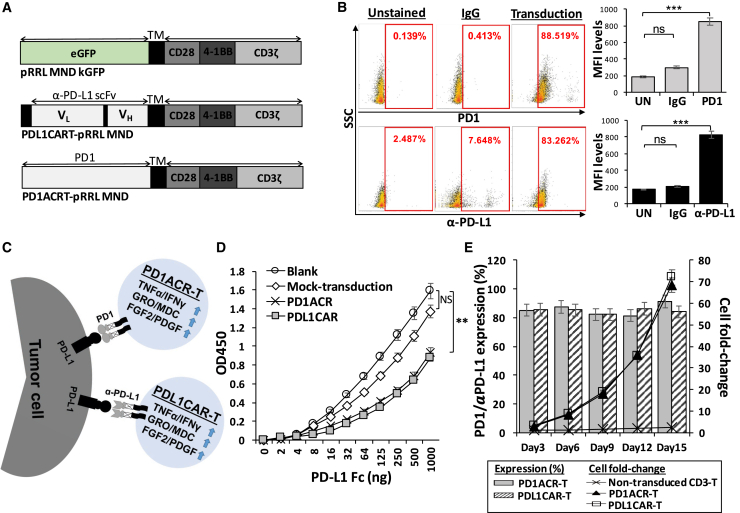
Characterization of Genetically Engineered PD1ACR and PDL1CAR T Cells (A) Schematic representation of a bicistronic lentiviral vector expressing ACR and CAR. (B) PD-1-specific ACR (PD1ACR) and αPDL1-specific CAR (PDL1CAR) expression levels on human T cells transduced with lentiviral particles were analyzed using flow cytometric antibodies anti-human PD-1 and anti-human IgG by detecting PD-1 and αPDL1 expression, respectively. Transduction efficiencies are shown inside each panel. (C) Additional features of PD1ACR-T and PDL1CAR-T cells that combat the PD-L1-expressing solid cancers. (D) A sandwich ELISA was performed to evaluate the binding ability of PD1ACR and PDL1CAR to human PD-L1 by using PD1ACR-, PDL1CAR-, and mock-transduced 293T cells. Untransduced 293T cells were employed as a negative control (blank) (∗∗p < 0.01). (E) Expression percentages plotted in bars and cell fold changes expressed in line plots of PD1ACR/PDL1CAR cells, presented as the mean ± SD, were derived from three independent healthy donors during a 2-week culture period in the stimulation of recombinant human PD-L1 Fc protein.

Interference of PD-1/PD-L1 interaction by the administration of blocking antibodies (nivolumab-αPD1 or avelumab-αPD-L1) could enhance the potency of immunotherapy targeting PD-L1-expressing pancreatic cancers (PaCs), and this strategy has reached phase II clinical trials.^8^ However, partial or no complete response (CR) was observed in patients, suggesting that the therapeutic effects of a naked antibody would not be potent enough for the curative treatment of PDAC. Therefore, in the present study, we investigated the CAR T cell strategy to disrupt the interaction of immune checkpoint inhibitors PD-1 and PD-L1 to effectively treat PD-L1-expressing PaCs. The detailed CAR engineering is illustrated in Figure 1, where the scFv region of human αPDL1 was used for the construction of PDL1CAR. Furthermore, to convert tumor PD-L1 to a ligand that transmits CD28 and 4-1BB costimulatory signals to effector T cells, we generated another combinatorial antigen receptor, PD-1-CD28-4-1BB activating chimeric receptor (PD1ACR). Accordingly, both types of PD-L1-based CARs, PD1ACR and PDL1CAR, were explored and compared for their potential in PaC treatment.

## Results

### Generation of PD1ACR- and PDL1CAR-T Cells by Lentiviral Vector Transduction

As shown in Figure 1A, lentiviral expression vectors encoding the PDL1-targeted activating chimeric receptor (ACR) and CAR, including PD1ACR and PDL1CAR, and negative control (mock) were constructed, which efficiently transduced human T cells to express PD-1, αPDL1, and EGFP individually by using the self-cleaving F2A peptide. For the PD1ACR plasmid (PD1ACRT-pRRL MND; Figure 1A, bottom panel), the extracellular domain sequence of human PD-1 (amino acids [aa] 1–170), including the signaling peptide, was designed to be fused to the transmembrane (TM) and intracellular sequences, whereas the scFv PD-L1 linked in-frame to the hinge domain of the CD8a molecule was used to obtain the PDL1CAR plasmid (PDL1CART-pRRL MND; Figure 1A, middle panel). Both vectors efficiently transduced human T cells. The expression of ACR and CAR in transduced T cells was demonstrated through PD-1 and αPDL1 expressions by using anti-PD-1 and anti-human immunoglobulin G (IgG), respectively. In flow cytometry analysis, the transduction efficiencies of PD1ACR (Figure 1B, top panel) and PDL1CAR (Figure 1B, bottom panel) were found to be approximately 88.5% and 83.2%, respectively. Moreover, the expressed mean fluorescence intensity (MFI) levels of PD-1 and αPDL1 expression significantly increased compared with their non-transduced and IgG controls (all p < 0.001; Figure 1B, right panel).

When ACR or CAR was expressed in effector T cells, it activated T cells effectively by costimulating CD28 and CD137 (4-1BB) upon engagement of PD-L1^+^ tumor cells (Figure 1C). To elucidate and compare recognition abilities between receptor/ligand and antigen/antibody in terms of the engineered ACR/CAR binding affinity, we transduced 293T cells with PD1ACR and PDL1CAR, respectively, with the lentiviral vector transduction used as a mock control and 293T cells without transduction used as a negative control (blank; Figure 1D). Sandwich ELISA results revealed that, compared with mock-transduced or non-transduced 293T cells (blank), PD1ACR- and PDL1CAR-transduced 293T cells could specifically bind the recombinant human PD-L1 Fc antigen, indicating that the PD-1 receptor and PD-L1-scFv contained in ACR/CAR could expectedly recognize the PD-L1 antigen. Moreover, the binding ability of both PD1ACR and PDL1CAR was similar to that of mock and blank controls (∗∗p < 0.01; Figure 1D).

To further investigate the proliferation capacity of genetically engineered T cells, we washed ACR and CAR T cells free of interleukin-2 (IL-2) by using 1× PBS after activation and transduction, and then cultured them in complete medium in the presence of 0.75 μg/mL recombinant human PD-L1 Fc protein. The cell counts and expression levels of ACR/CAR T cells were monitored at different time points for an additional 2 weeks (Figure 1E). Results obtained using T cells from three different donors were consistent in this assay. PD-1-expressing PD1ACR T cells and αPDL1-expressing PDL1CAR T cells increased by more than 50-fold and were highly enriched for ACR/CAR cells compared with non-transduced CD3 T cells during prolonged culture following PD-L1 antigen stimulation (Figure 1E). In addition, the surface expression frequency of both ACR and CAR on T cells remained stable at approximately 80% over the entire expansion period, suggesting that a costimulatory signal is required for ACR/CAR enrichment.

### Co-expression of Activation or Exhaustion Markers in Transduced CAR T Cells and PD-L1 Expression in Human PaC Cells

**Figure 2:**
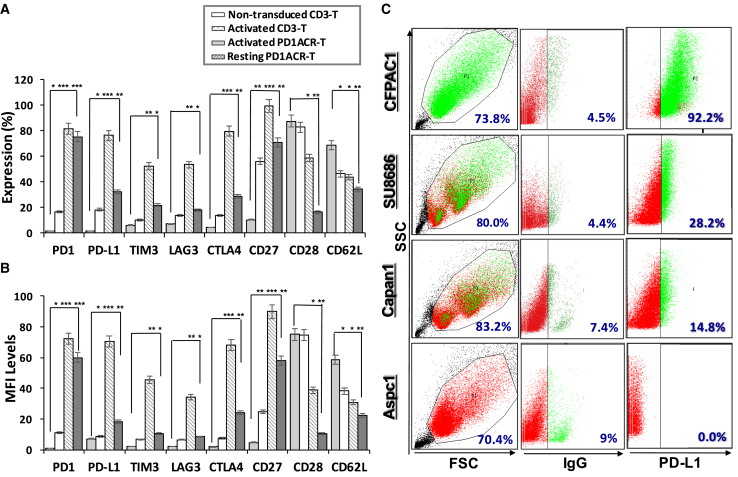
Co-expression of Activated and/or Inhibited Markers in Transduced CAR T Cells and the Expression of PD-L1 in Human Pancreatic Cancer Cell Lines (A and B) T cell activation markers (CD27, CD28, and CD62L) and inhibitory markers (PD-1, PD-L1, TIM3, LAG3, and CTLA4) in CARs were investigated through flow cytometry for (A) expression (%) and (B) MFI levels gated on the positive cells; data shown are the average value from three independent experiments (∗p < 0.05, ∗∗p < 0.01, ∗∗∗p < 0.001). (C) Representative flow cytometry dot plots demonstrating PD-L1 expressions among the four pancreatic cancer cell lines, including CFPAC1, SU8686, Capan1, and Aspc1. Data of at least three independent experiments are presented.

We generated human CAR constructs encoding PD-1 and/or αPD-L1 (both are PD-L1-targeted CARs). Transduced T cells expressed CAR on the surface (Figure 1C), and we also investigated co-expressions of T cell activation markers, including CD27, CD28, and CD62L, as well as T cell inhibitory markers, including PD-1, PD-L1, TIM3, LAG3, and CTLA4, at different statuses of T cells by using a flow cytometric analysis. As presented in Figure 2, activated PD1ACR-T cells showed significantly increased percentage (%; Figure 2A) and MFI levels (Figure 2B) of their activation markers CD27, CD28, and CD62L compared with the resting status of PD1ACR-T cells and non-transduced CD3 T cells (∗∗p < 0.01, ∗∗∗p < 0.001; Figures 2A and 2B). Moreover, activated PD1ACR-T cells significantly co-expressed inhibitory markers, including PD-L1, TIM3, LAG3, and CTLA4, compared with other controls (∗p < 0.05, ∗∗p < 0.01, ∗∗∗p < 0.001; Figures 2A and 2B).

We further detected PD-L1 protein and messenger RNA (mRNA) expression levels in four different PaC cell lines (CFPAC1, SU8686, Capan1, and Aspc1) by using flow cytometry, western blot, and quantitative reverse transcription polymerase chain reaction (qRT-PCR) analyses. Flow cytometry revealed that the level of PD-L1 was highest in the CFPAC1 surface among the four PaC cell lines (Figure 2C). Western blotting results revealed that intracellular PD-L1 expression did not vary dramatically among the four PaC cell lines (Figure S1A). Likewise, PD-L1 mRNA was significantly higher in the CFPAC1 cell line (∗p < 0.05) than in other cells (Figure S1B). Judging from the surface expression levels of PD-L1 on the four representative PaC cell lines, we chose CFPAC1 (a high-PD-L1-expressing cell line) and Capan1 (a low-PD-L1-expressing cell line) for further investigation.

### *In Vitro* Cytotoxicity of Genetically Modified T Cells Redirected to PD-L1 on Human PaC Cells

**Figure 3:**
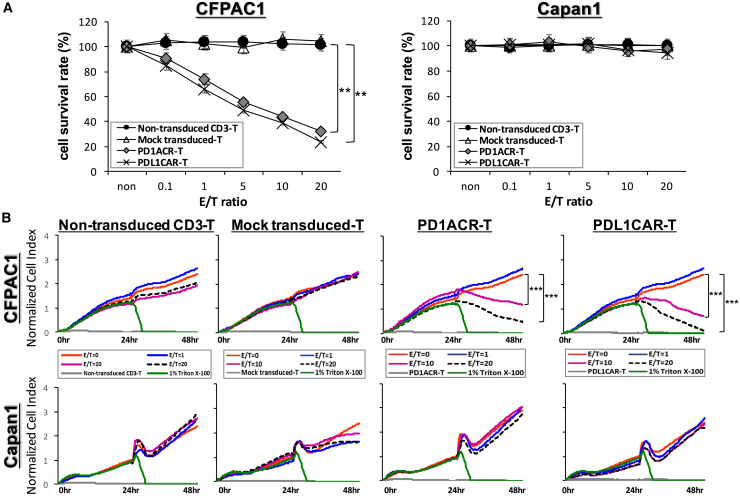
A Specific Suppression of PD-L1-Expressing CFPAC1 Cells by Both PD1ACR-T and PDL1CAR-T Cells *In Vitro* (A) Standard 24-h cytotoxicity activities were performed using MTT assays with at least three replicates (n > 3) with increasing effector (PD1ACR-T and PDL1CAR-T cells) concentrations to target E:T ratios of 0, 0.1, 1, 5, 10, and 20 against cancer cell lines, CFPAC1 and Canpan1. Cytotoxic activities were compared with non-transduced CD3 T cell-treated cells, and mock-transduced T cell-treated cells served as the controls of both chimeric modified T cells (n > 3; ∗∗p < 0.01). (B) Real-time monitoring of cytotoxic activities was compared with non-transduced CD3 T cell-treated and mock-transduced T cell-treated cells, and Triton X-100 (1%)-treated cells served as the positive control. Real-time monitoring of PD1ACR-T and PDL1CAR-T cell-treated cells induced growth inhibition of PD-L1-expressing CFPAC1 cells compared with PD-L1 low-expressing Capan1 cells (n > 3; ∗∗∗p < 0.001, respectively), as observed using the x-CELLigence system. Data are presented as the mean ± SD of three independent experiments.

To determine whether T cells targeting PD-L1 could specifically recognize and kill PD-L1-positive PaC cells, we performed cytotoxicity assays by incubating genetically modified T cells and PD1ACR-T and PDL1CAR-T cells with the two selected PaC cell lines (CFPAC1 and Capan1) at effector/tumor (E:T) ratios of 0.1, 1, 5, 10, and 20. Both PD1ACR-T and PDL1CAR-T cells could efficiently lyse PD-L1-high CFPAC1 cells, but not PD-L1-low Capan1 cells, as observed in the 3-(4,5-dimethylthiazol-2-yl)-2,5-diphenyltetrazolium bromide (MTT) assay (∗∗p < 0.01; Figure 3A), whereas control effector cells (mock-transfected) and non-transduced CD3 T cells could not initiate specific lysis on either cell line.

A real-time cell monitoring system, xCelligence System,^27^ was used to further investigate the cytotoxic effect of PD1ACR-T and PDL1CAR-T cells toward PD-L1-expressing pancreatic tumor cells. For the first 24 h, tumor cells grew almost directly proportional to the time of culture. The addition of genetically modified T cells (mock-transfected and non-transduced CD3 T cells), along with the replacement of old medium at 24 h with the E:T ratios of 1, 10, and 20, caused a slight decrease in the cell index (CI) of control cells, which was probably related to stress. The addition of E:T ratios at 10 and 20 of PD1ACR-T or PDL1CAR-T cells alone resulted in an abrupt decrease in impedance, and CI values were significantly lower in PD-L1-high CFPAC1 cells compared with PD-L1-low Capan1 control cells (∗∗∗p < 0.001, respectively; Figure 3B) and those treated with corresponding E:T ratios of mock-transfected and non-transduced CD3 T cells, whereas Triton X-100-treated cells all died and served as the positive control (Figure 3B). These results were consistent with MTT data (Figure 3A), confirming that modified PD1ACR-T or PDL1CAR-T cells treated alone retained significant cytotoxic activities toward PD-L1-positive pancreatic tumors specifically.

### ACR T and CAR T Cells Redirected to PD-L1 Significantly Suppress the Tumorigenesis of Subcutaneous CFPAC1 Xenografts

**Figure 4:**
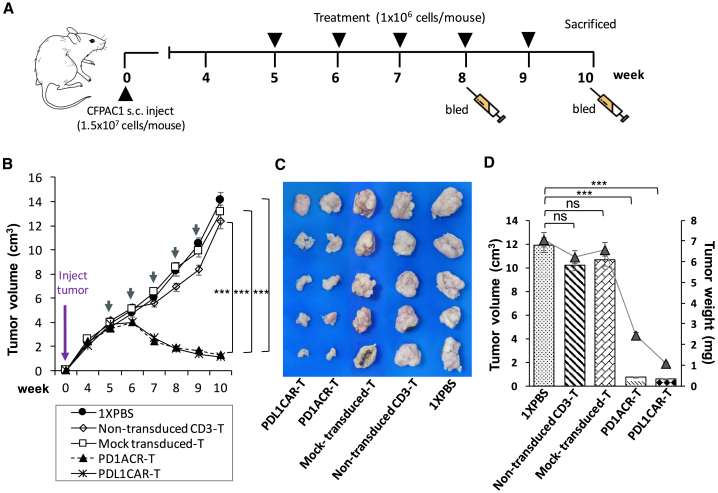
Potent Growth Suppression on Established Subcutaneous (s.c.) CFPAC1 Xenografts by PD-L1-Targeted CAR T Cells *In Vivo* (A) A detailed treatment schedule of *in vivo* study was shown. (B) Growth curve of CFPAC1 xenografts treated with indicated T cells or 1× PBS. At the endpoint (week 10), residual tumors treated with PD1ACR-T and PDL1CAR-T cells were significantly smaller than those in control groups, respectively (n = 6 per group; ∗∗∗p < 0.001). (C and D) The endpoint dissection of treated mice. Tumor masses (C), mean tumor volume (mm^3^) plotted in bar graph, and mean tumor weight (mg) expressed in line plots (D) from each treated mouse group (n = 6 per group; ∗∗∗p < 0.001, respectively).

To explore the antitumor activities of both PD1ACR-T and PDL1CAR-T cells toward PD-L1-expressing tumors, we used nude mice bearing established subcutaneous (s.c.) CFPAC1 xenografts. As described in Figure 4A, CFPAC1 cells (1.5 × 10^7^) s.c. implanted into mice grew to discernible tumor masses (approximately 4,000 mm^3^ in volume) for 5 weeks before initiating immunotherapy. To test the antitumor efficacy, we divided experimental mice into five groups (n = 6 per group), which received PD1ACR-T cells, PDL1CAR-T cells, mock-transduced-T cells, non-transduced CD3 T cells, or 1× PBS treatment. The potent antitumor effect was observed in mice treated with both PD1ACR-T and PDL1CAR-T cells, whereas other genetically modified T cells or non-transduced CD3 T cells did not suppress tumor growth (Figures 4B–4D). At the experimental endpoint (week 10), all mice treated with PD1ACR-T cells or PDL1CAR-T cells had significantly decreased tumor volume (n = 6 per group, ∗∗∗p < 0.001; Figure 4B), tumor size (Figure 4C), and tumor weight (n = 6 per group, ∗∗∗p < 0.001; Figure 4D), whereas all mice in the control groups had large tumors. These results suggested that both PD1ACR-T cells and PDL1CAR-T cells confer strong antitumor activities and could specifically eliminate CFPAC1 cells *in vivo*.

In mice treated with a different schedule (Figure S2A), CFPAC1 cells (1 × 10^7^) were s.c. implanted in mice and allowed to form tumor masses (approximately 800 mm^3^) for 7 weeks before initiating immunotherapy. Subsequently, PD1ACR-T or PDL1CAR-T cells were systemically administered every week for 5 consecutive weeks. Consistent with previous results, a significant and long-term cessation of CFPAC1 tumor growth was achieved compared with control mice in which the tumor size increased over time (n = 4 per group; Figure S2B). At week 13, tumor volumes and tumor weight had significantly decreased in both groups of treated mice compared with control mice (n = 4 per group; ∗p < 0.05; Figures S2C and S2D).

**Figure 5:**
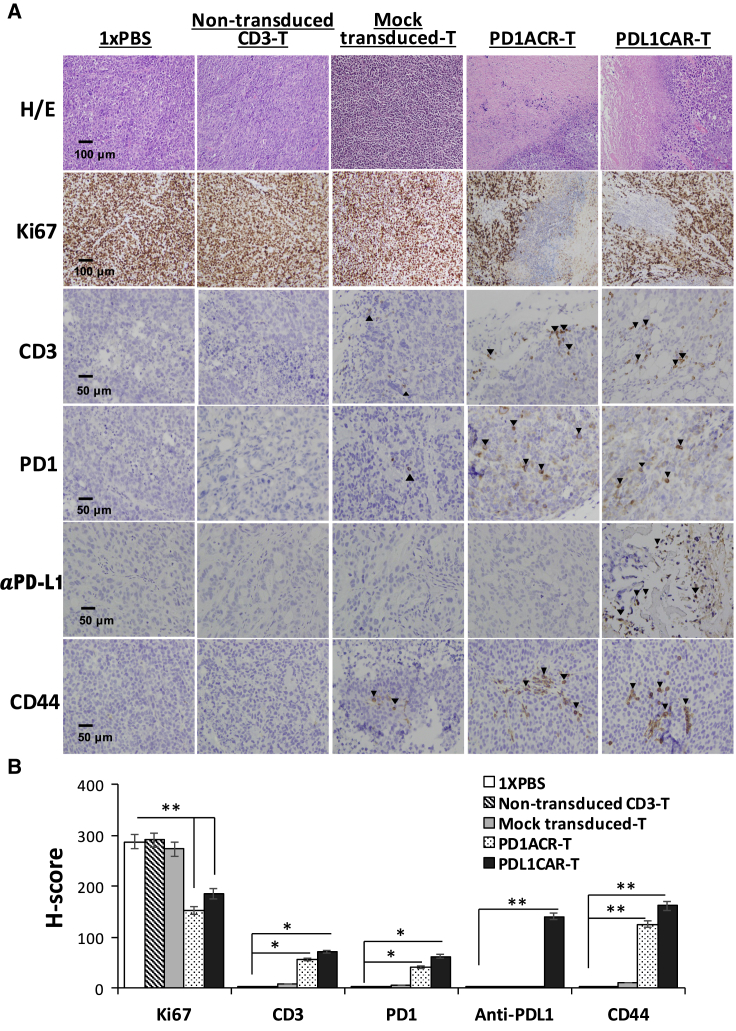
PD-L1-Targeted CAR T Cells Could Be Located in CFPAC1 Tumors Tumors were collected from mice bearing CFPAC1 subcutaneous (s.c.) xenografts treated with PDL1CAR-T cells, PD1ACR-T cells, mock-transduced T cells, non-transduced CD3 T cells, or 1× PBS. (A) Formalin-fixed, paraffin-embedded tumor sections were consecutively cut and stained for H&E, human Ki67, CD3, PD-1, anti-PD-L1, and CD44 expressions (black arrowheads). Images were taken using a microscope (BX50; Olympus, Tokyo, Japan) and camera (DP22) under 400× or 200× original magnification. Individual scale bars are shown. (B) The average H score for each marker and comparison between groups are shown (∗p < 0.05; ∗∗p < 0.01).

The persistence of transferred T cells *in vivo* is highly correlated with tumor regression.^28^ Therefore, we also detected the infiltration of human-modified T cells in the tumor tissue of mice bearing s.c. established CFPAC1 xenografts at the endpoint after T cell infusion. The persistence of human T cells was confirmed by immunostaining of the sections of CFPAC1 tumors treated with both PD1ACR-T and PDL1CAR-T cells. Results revealed that human CD3^+^ T cells, PD-1^+^ T cells, αPD-L1^+^ T cells, and CD44^+^ T cells had accumulated in residual tumors after intravenous (i.v.) T cell administration (Figures 5 and S3), whereas no specific staining could be detected in the sections of tumors treated with mock-transduced T cells, non-transduced CD3 T cells, or 1× PBS. Moreover, we examined tumor cell proliferation by performing Ki67 staining in dissected tumor tissues. Tumor sections from control mouse groups showed a much higher Ki67 index than did those from both genetically modified T cell-treated mice. This was also confirmed by the H&E staining of tumor sections, in which necrotic areas were significantly increased in genetically modified T cell-treated tumor sections compared with the control (Figures 5 and S3).

### ACR/CAR T Cells Redirected to PD-L1 Significantly Eradicate Tumor and Extend the Overall Survival of Orthotopic CFPAC1 Models

**Figure 6:**
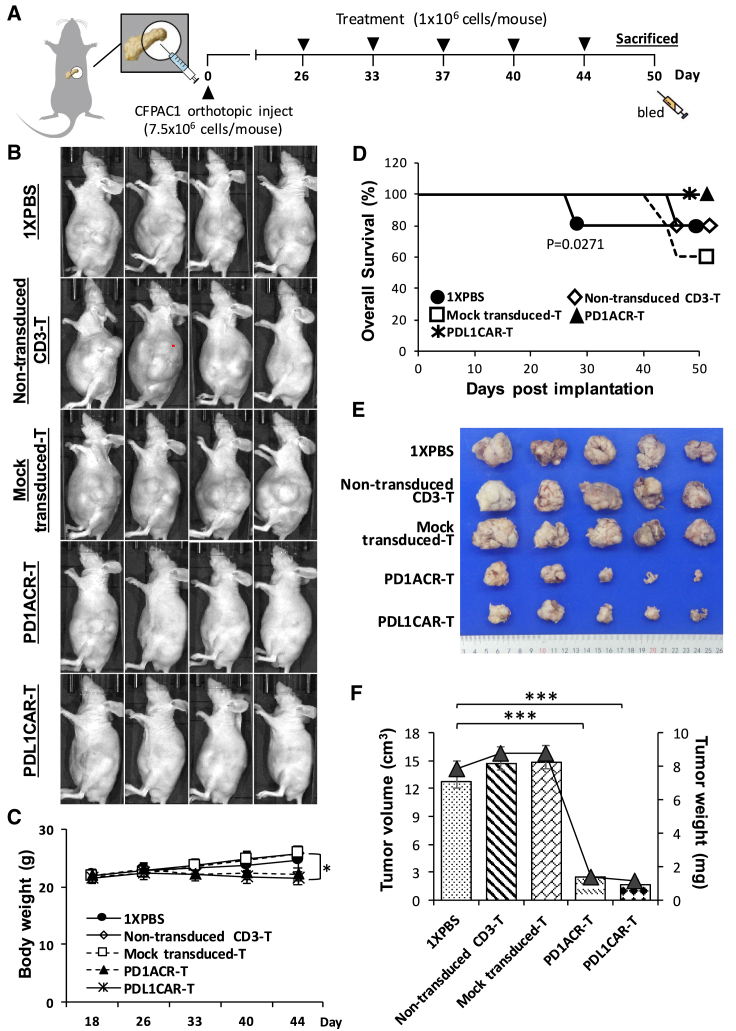
Potent *In Vivo* Growth Suppression in the Established Orthotopic CFPAC1 Model by PD-L1-Targeted CAR T Cells (A) A detailed treatment schedule of the orthotopic model. (C) Mean body weight curve of individual CFPAC1 orthotopic models over time. At the endpoint (day 50), mouse groups treated with PD1ACR-T and PDL1CAR-T cells were significantly mitigated compared with those in control groups, respectively (n = 6 per group; ∗p < 0.05). (B and D–F) The endpoint dissection of treated mice. Whole-body appearance (B), overall survival curve (D), pancreatic tumor masses (E), mean tumor volume (mm^3^) plotted in the bar graph, and mean tumor weight (mg) expressed in line plots (F) from each treated mouse group (n = 6 per group; ∗∗∗p < 0.001).

To further explore the anti-tumor potential of ACR/CAR T cell immunotherapy in PDAC, we included an orthotopic tumor model (Figure 6) to test the efficacy of PD1ACR-T and PDL1CAR-T cells individually. The treatment schedule (Figure 6A) involved the orthotopic implantation of CFPAC1 cells (7.5 × 10^6^) into the mouse pancreas until they grew to discernible tumor masses (approximately 7.34E+8 in *In Vivo* Imaging System [IVIS] signal intensity) for 26 days before initiating immunotherapy. Experimental mice were then randomly divided into five groups (n = 6 per group), which received one of the following treatments: PD1ACR-T cells, PDL1CAR-T cells, mock-transduced T cells, non-transduced CD3 T cells, or 1× PBS treatment. We observed a significantly increased mean body weight in the three control mouse groups compared with the group consisting of ACR/CAR T cell-treated mice (∗p < 0.05; Figure 6C) due to the obvious tumor mass enlargement and ascites formation in controls. Next, the overall survival rate was 80% and 60% for mice administered 1× PBS/non-transduced CD3 T cells and mock-transduced T cells, respectively, in the orthotopic models and almost 100% for mice treated with PD1ACR-T and PDL1CAR-T cells (p = 0.0271; Figure 6D). Because ascites affects the bioluminescence imaging photon flux, bioluminescence imaging measurements did not recapitulate tumor growth as observed using the IVIS imaging system^29^ in our orthotopic tumor models. Therefore, at the experimental endpoint (day 50), all mice treated with PD1ACR-T or PDL1CAR-T cells had significantly decreased tumor volume in whole-body appearance (Figure 6B), dissected tumor size (Figure 6E), and measured tumor volume (cm^3^) and tumor weight (mg) (∗∗∗p < 0.001; Figure 6F), whereas all mice in the control groups had large tumors. These results again suggested that both PD1ACR-T and PDL1CAR-T cells confer strong antitumor activities and increased median survival by specifically eliminating CFPAC1 cells in orthotopic tumor models.

**Figure 7:**
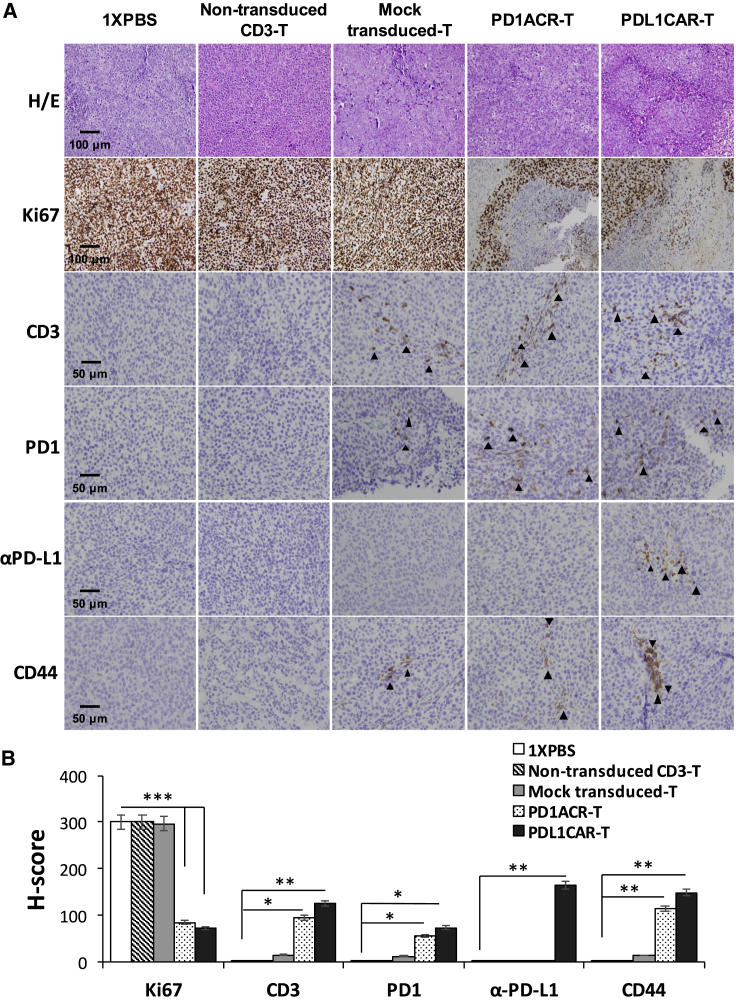
PD-L1-Targeted ACR/CAR T Cells Could Be Located in Orthotopic CFPAC1 Tumors Tumors were collected from mice bearing orthotopic CFPAC1 xenografts treated with PDL1CAR-T cells, PD1ACR-T cells, mock-transduced T cells, nontransduced-CD3 T cells, or 1× PBS. (A) Formalin-fixed, paraffin-embedded tumor sections were consecutively cut and stained for H&E, human Ki67, CD3, PD-1, anti-PD-L1, and CD44 expressions (black arrowheads). The images were taken with a microscope (BX50, Olympus, Tokyo, Japan) and camera (DP22) under 400× or 200× original magnification. Individual scale bars are shown. (B) The average H score for each marker and between-group comparisons are shown (∗p < 0.05; ∗∗p < 0.01).

We also validated the infiltration of ACR/CAR T cells in the tumor tissues of mice bearing orthotopic established CFPAC1 xenografts at the endpoint following T cell infusion. Results were similar to those of s.c. xenograft models: human CD3^+^ T cells, PD-1^+^ T cells, αPD-L1^+^ T cells, and CD44^+^ T cells accumulated in residual tumors following i.v. T cell administration, whereas no specific cells were detected on staining in the sections of tumors treated with mock-transduced T cells, non-transduced CD3 T cells, or 1× PBS, and only αPD-L1^+^ T cells were detected in the PDL1CAR-T cell-treated mouse group (Figure 7). Moreover, the Ki67 index (a marker of tumor cell proliferation) was much higher in tumor sections from control mouse groups than in those from ACR/CAR T cell-treated mice (Figure 7).

### Genetically Engineered T Cells Secreted Cytokines with Enhanced Antitumor Function *In Vivo*

**Figure 8:**
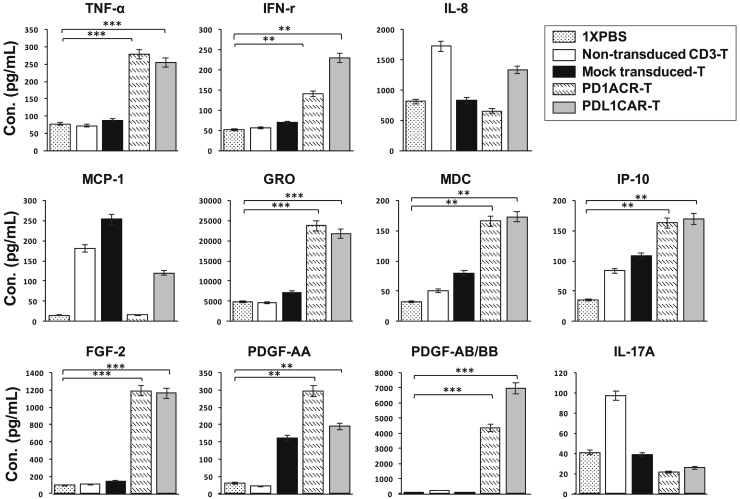
Genetically Engineered T Cells Secreted Cytokines with Enhanced Antitumor Function in Xenograft PaC Models Serum samples from each treated mouse group during the treatment period were subjected to cytokine analysis by using the human cytokine/chemokine Magnetic Bead Panel protocol from Milliplex. Shown are the concentrations (pg/mL) of selected proinflammatory cytokines (TNF-α, IFN-γ, and IL-8; upper panels), chemokines (MCP-1, GRO, MDC, and IP-10; middle panels), and growth factors, as well as those that regulate T cell polarization/differentiation (FGF-2, PDGF-AA, PDGF-AB/BB, and IL-17A; lower panels). Cytokines/chemokines in serum samples with undetectable levels were excluded (data not shown). Data are presented as the mean ± SD of three independent experiments.

Activation of genetically modified T cells upon antigen encountering was accompanied by the release of cytokines and/or chemokines in circulation. We then detected 41 serum cytokine/chemokine levels from mice treated with PD1ACR-T cells, PDL1CAR-T cells, and control T cells, including mock-transduced T cells (mock), non-transduced CD3 T cells, and 1× PBS (shown as control) using the human Cytokine/Chemokine Magnetic Bead Panel protocol from Milliplex (Figure 8). Therefore, 30 of 41 measured cytokines with below-detectable levels were excluded from the statistical analysis (data not shown). The other 11 cytokines (tumor necrosis factor alpha [TNF-α], interferon (IFN)-γ, IL-8, monocyte chemotactic protein-1 [MCP-1], growth-regulated oncogene (GRO), macrophage-derived chemokine [MDC], Interferon gamma-induced protein 10 (IP-10), fibroblast growth factor-2 [FGF-2], platelet-derived growth factor [PDGF]-AA, PDGF-AB/BB, and IL-17A) were categorized into four groups based on distributions among those treated mouse groups: proinflammatory cytokines, chemokines, growth factors, and regulatory cytokines.

Analyses of the proinflammatory cytokines TNF-α and IFN-γ in the serum of mice treated with PD1ACR-T and PDL1CAR-T cells compared with the other three treated controls (Figure 8, top panels) revealed that the encountered PD-L1 tumor antigen led to a significant (∗∗∗p < 0.001 and ∗∗p < 0.01, respectively) increase in the levels of both cytokines. In addition, we observed significantly increased serum chemokine (GRO, MDC, and IP-10) levels in mice treated with PD1ACR-T and PDL1CAR-T cells relative to controls without encountering the PD-L1 tumor antigen (∗∗∗p < 0.001, ∗∗p < 0.01, and ∗∗p < 0.01, respectively; Figure 8, middle panels). Furthermore, serum growth factor (FGF-2, PDGF-AA, and PDGF-AB/BB) levels were significantly increased in mice treated with both ACR/CAR T cells (∗∗∗p < 0.001, ∗∗p < 0.01, and ∗∗∗p < 0.001, respectively; Figure 8, bottom panels) compared with treated controls, whereas cytokines that are known to regulate (IL-17A) T cell polarization and differentiation were similar in the serum of all mouse groups (Figure 8, bottom panels). These data indicate that the robust induction of systemic inflammatory cytokines is associated with the ACR/CAR T cell activation following antigen encountering, thereby significantly enhancing antitumor activities.

**Figure 9:**
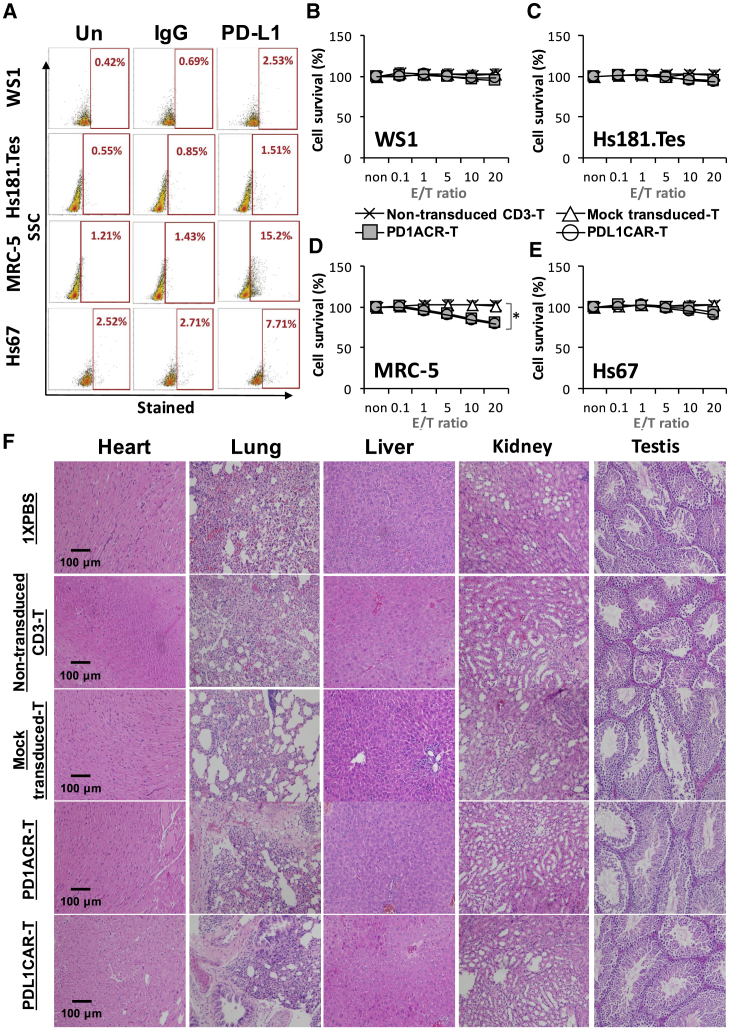
Safety Evaluation of CAR T Cell Therapy (A) Flow cytometry reveals surface PD-L1 levels in different normal human cell lines. (B–E) Both PD1ACR-T and PDL1CAR-T cells show no cytolytic activity against healthy WS1 (B) and Hs181.Tes (C) cells. Both MRC-5 (D) and Hs67 (E) cell lines are sensitive to PD1ACR-T and PDL1CAR-T cells *in vitro* (*p< 0.05). Data are presented as the mean ± SD of three independent experiments. (F) H/E staining displays no obvious off-target toxicity against major mice organs. Original magnification, 200X. Scale bars, 100 um.

Finally, to further evaluate that PD1ACR-T and PDL1CAR-T cells have no cytotoxic activity against healthy tissues, WS1, Hs181.Tes, MRC-5, and Hs67 healthy cell lines were used as targets for *in vitro* lytic assays. The expression of PD-L1 on healthy cells was investigated through flow cytometry (Figure 9A); no significant cytotoxic activity was observed against healthy WS1, Hs181.Tes, and Hs67 cells (Figures 9B, 9C, and 9E), whereas in MRC-5 cells, which expressed low levels of PD-L1 (at approximately 15%), both PD1ACR-T and PDL1CAR-T cells exhibited low levels of cytotoxicity (∗p < 0.05; Figure 9D). To investigate the potential toxicity of ACR/CAR T cells, we excised orthotopic murine organs, including the heart, lung, liver, kidney, and testis, and examined them histologically; no morphological changes caused by off-target toxicity were noted (Figure 9F).

## Discussion

The expression of PD-L1 on many primary tumor cells and immunosuppressive cells (e.g., myeloid-derived suppressor cells) within the TME makes the PD-1/PD-L1 axis an attractive target for the development of immunotherapies. In this study, we developed PD-L1-targeted ACR/CAR T cells by using third-generation CARs containing additional signaling domains, including CD28, CD137, and OX40.^23^^,^^24^ Both types of PD-L1-targeted ACR/CAR T cells could specifically destroy PaC cell-associated PD-L1 *in vitro* and in the tumor xenograft/orthotopic mouse models. Our analyses revealed that the cytotoxic activities of both engineered T cells toward PaC cells were significantly associated with their targeting specificity. Also, compared with mock transduction T cell, non-transduced CD3 T cell, or 1× PBS-treated control mouse groups, both ACR/CAR T cell-treated mouse groups preserved the similar abilities of TME infiltration, cytokine/chemokine induction, tumor elimination, and long-term tumor-free survival in a tumor xenograft mouse model. Based on these results, we rationalized the extension of our PD-L1-targeted ACR/CAR T cell-based immunotherapy approach to treat human PDAC.

PD1ACR/PDL1CAR T cells secrete TNF-α and INF-γ upon stimulation with PD-L1^+^ tumor cells and display potent cytolytic capacity *in vitro*^30^ against PD-L1^+^ PaC cells. Consistently, the production of proinflammatory cytokines and cytolytic capacity by PD1ACR- and PDL1CAR-T cells substantially increased after co-culture with PD-L1^+^ PaC cells compared with mock-transduced T cells. Mechanisms accounting for increased effector function by 4-1BB and CD28 costimulated CAR T cells *in vitro* to have their ability to resist antigen-induced cell death (AICD).^31^^,^^32^ These findings are also consistent with the finding of ACR/CAR T cells exhibiting *in vivo* antitumor effects on the highly invasive CFPAC1 xenograft model of human PaC cells, where tumor regression was associated with enhanced T cell activation *in vivo*. Furthermore, the T cell polarized regulatory cytokine IL-17A did not show any significant difference among the treated mouse groups, probably because of the controversial source of this cytokine at the site of inflammation,^33^ which requires further investigation.

The efficacy of our PD-L1-targeted CAR T cell treatment for PaC may also be influenced by surface PD-L1 expression levels and their binding affinity in cancer cells, as well as in the same tumor model. Thus far, clinical success using CAR-transduced cells for the treatment of solid tumors has been limited. Clinical trials based on CAR targeting single antigens, such as mesothelin (MSLN),^34^ prostate stem cell antigen (PSCA),^35^ carcinoembryonic antigen (CEA),^36^ mucin 1,^37^ and human epidermal growth factor (EGF) receptor 2 (HER2, also known as ERBB2),^38^ were also investigated for human PDAC; results found mixed efficacy, thus confirming a fundamental issue with CAR T cell therapy: a lack of ideal single-antigen targets. Another major obstacle for CAR T cell therapies in solid tumors, such as human PDAC, is the immunosuppressive TME,^39^ which may greatly hamper targeted immunotherapy clinically. Therefore, reports recommended that combining CAR T cells with other therapies, such as CTLA-4 and PD-1 inhibitors, could improve antitumor effects. Specifically, the PD1/PD-L1 interaction suppresses effector T cell function; tumors appear to take advantage of this pathway and express PD-L1, thereby presenting potential for an adoptive T cell immunotherapeutic strategy. Xie et al.^40^ demonstrated that PD-L1-targeted CAR T cells exerted a mild cytotoxic effect against a PD-L1-positive lung cancer cell line *in vitro* and did not attack nearby T cells with low PD-L1 expression. Subsequently, enhanced antitumor function was demonstrated using interrupted PD-1 signaling in combination with adoptive CAR T cell therapy, such as PSCA-BBZ/PD1CD28 CAR-T^41^ and bi-specific Trop2/PD-L1 CAR-T^42^ targeted against prostate cancer and gastric cancer, respectively, which claimed to improve the killing effect of CAR T cells in solid tumors. Whereas our strategy of utilizing PD-L1-targeted CARs has the advantages of not only eliminating PD-L1-overexpresing cancer cells but also blocking immune checkpoint (PD-1/PD-L1 axis), thereby significantly enhancing T cell antitumor activity. In addition, we compared the effects of PD1ACR-T to PDL1CAR-T cytotoxicity in terms of their ability to bind to PD-L1, confirming that both ACR/CAR T cells retained a similar antitumor function.

Systemic administration of checkpoint blockades, anti-PD-1 and/or anti-PD-L1, can frequently result in immune-related adverse events (IRAEs).^19^^,^^43^^,^^44^ Because of poor trafficking after i.v. injection, local instillation of CARs is being explored; clinical trials (ClinicalTrials.gov: NCT02414269 and NCT01818323)^45^ are evaluating the merits of site-specific administration—systemic versus regional versus intratumoral administration—of CAR T cells in solid tumors. A potential limitation is that local instillation is often more technically challenging than simple i.v. administration. Given that CAR T cells traffic to and expand at the tumor site, delivery of PD-L1-targeted CARs is primarily localized to this tumor area in our xenograft and orthotopic models. Given the translational intent of our study, a potential study limitation of the present study is that our immunodeficient mice precluded the consideration of the contributions of other potentially critical cell types, such as endogenous myeloid-derived suppressor cells or macrophages that might have high levels of PD-L1 that could interact with T cells.^46^ Therefore, we speculate that the strategy presented in the present study, that is, using localized and not systemic delivery, can reduce IRAEs associated with systemic checkpoint blockade; this is also being investigated in our ongoing studies.

The present proof-of-concept study provides support for the strategy of using armed CAR T cells for targeted delivery of immunomodulatory scFv to TME not only for the PD-1/PD-L1 axis but also for expanding to other immune-modulating molecules, such as LAG-3,^47^ TIM-3,^48^ and CTLA4,^49^ or a combination thereof. Our strategy can potentially improve the clinical outcome in response to CAR T cell therapy and improve the safety of immune checkpoint blockade therapy.

## Materials and Methods

### Ethics, Consent, and Permission

The Taipei Medical University-Joint Institutional Review Board (TMU-JIRB) approved the study (protocol no.: N201605059, June 10, 2016). Informed consent was obtained from each healthy volunteer. The research, including biohazards, biological agents, toxins, materials, and reagents, followed the standard biosafety regulations and was reviewed and approved by the Institute’s Environmental Protection and Biological Safety Committee (G-104-078, January 4, 2016) before the project started.

### Cell Lines

Human PaC cell lines (CFPAC1, SU8686, Capan1, and Aspc1), human normal cell lines (WS1, Hs181.Tes, MRC-5, Hs67), and 293T cells were purchased from American Type Culture Collection (ATCC, Manassas, VA, USA) and grown in Dulbecco’s modified Eagle’s medium (Life Technologies, Carlsbad, CA, USA) supplemented with 10% FBS at 37°C with 5% CO_2_ incubation. All cell lines were tested for species identification and mycoplasma detection, and authentication was confirmed using short tandem repeat (STR) profile analysis at ATCC.

### Construction of Lentiviral Vectors Encoding CAR and ACR

The nucleotide sequence encoding the human PD-1 and human anti-PD-L1 (αPDL1) scFv antibody in the V_L_-V_H_ orientation was codon optimized and synthesized (Life Technologies). The shuttle plasmid pRRLMNDkGFP lentiviral vector was used in the construction. As shown in Figure 1A, the αPDL1 CAR (third generation) comprising the scFv PD-L1 linked in-frame to the hinge domain of the CD8α molecule (GenBank: NM_001145873.1) was fused to the TM region of the human CD28 molecule (GenBank: NM_006139.3) and the intracellular signaling domains CD137 (4-1BB) (GenBank: NM_001561.5) and CD3ζ (GenBank: NM_198053.2) in tandem. Next, the αPDL1 scFv was replaced by the human PD-1 sequence (UniProt: Q15116), similar to a murine system,^50^ to generate the PD1ACR-T lentiviral vector.

### Lentivirus Production

Human 293T cells were seeded at 9 × 10^6^ per 15-cm dish prior to 24 h of transduction. All plasmid DNA was purified using the Endo-Free Maxi prep kit (QIAGEN, Valencia, CA, USA). The 293T cells were transfected with 7.5 μg of the empty vector (mock) or the recombinant expression vector in addition to the 4.5 μg of packaging plasmid psPAX2 and 3 μg of the vesicular stomatitis virus envelope plasmid pMD2G by using a calcium phosphate transfection system.^51^ The viral supernatant was harvested at 48 h after transfection.

### Isolation, Activation, and Transduction of Human CD3^+^ T Cells

Peripheral blood mononuclear cells (PBMCs) were derived from healthy human donors. Primary human CD3^+^ T cells were isolated from PBMCs by positive selection using REAlease CD3 MicroBead kit (Miltenyi Biotec, Bergisch Gladbach, Germany). Isolated CD3^+^ T cells were stimulated for 24 h with recombinant human IL-2 (100 U/mL; PeproTech, Rocky Hill, NJ, USA) plus anti-CD3 (10 ng/mL; eBioscience, Pittsburgh, PA, USA) antibodies in accordance with the manufacturer’s instructions. CD3^+^ T cells were then transduced with the lentiviral vector at a multiplicity of infection of 16 U/cell. The transfected T cells were cultured at 8 × 10^5^ cells/mL in the presence of recombinant human IL-2 (300 IU/mL) every other day. Genetically modified T cells were isolated using a flow sorter (FACSAria III; BD Biosciences, San Jose, CA, USA) prior to being used for functional assays.

### Sandwich ELISA

A sandwich ELISA was performed to evaluate the binding ability of both PD1ACR and PDL1CAR to PD-L1, as described earlier.^52^ In brief, 96-well plates were seeded with transduced 293T cells (PD1ACR, PDL1CAR, and mock). Untransduced 293T cells (blank) were used as a negative control. Each well was washed, and recombinant human PD-L1 Fc antigens were added (R&D Systems, Minneapolis, MN, USA) at different dilutions. Next, supernatants were collected and added to another 96-well plate, which was preliminarily coated with an anti-IgG (Fc) antibody, followed by the addition of a biotinylated IgG (Fc) detector antibody and a detection conjugate (Aviva Systems Biology Corporation, San Diego, CA, USA). After substrate detection, the optical density at 450 nm (OD450) was measured with an automatic microplate reader (BioTek, Winooski, VT, USA).

### Western Blotting

Human PaC cells (CFPAC1, SU8686, Capan1, and Aspc1) were seeded and collected for western blotting. Cell lysates were denatured and electrophoresed through SDS-PAGE, as previously described.^53^ Primary antibodies against PD-L1 and α-tubulin were purchased from Novus Biologicals (Littleton, CO, USA), and α-tubulin was taken as a loading control. The appropriate secondary antibodies were used to detect proteins, as previously described.^53^ An ImageQuant LAS 4000 analyzer (GE Healthcare Life Science, Pittsburgh, PA, USA) was used to quantify the protein expression.

### Flow Cytometry

Cancer cell lines and CAR-modified T cells were prepared according to the manufacturer’s instructions. The following monoclonal antibodies were obtained from BD Biosciences: phycoerythrin (PE)-mouse anti-human PD-1 (CD279), allophycocyanin (APC)-mouse anti-human PD-L1 (CD274), BB515-mouse anti-human IgG, V450-mouse anti-human CD62L, PE-mouse anti-human CTLA4 (CD152), APC-mouse anti-human CD27, APC-mouse anti-human CD28, PE-mouse anti-human TIM-3 (CD366), PE-mouse anti-human LAG-3 (CD223), and their IgG controls. After preparation, CAR-modified T cells and tumor cell lines were confirmed using the FACSCanto II flow cytometer and analyzed using CellQuest Pro (BD Biosciences) software.

### Cytotoxicity Assays *In Vitro*

MTT (Sigma-Aldrich, St. Louis, MO, USA) cytotoxicity assays were performed in 96-well plates. Tumor cells (10^4^ cells/well) were plated in triplicate wells. After 24 h, at the indicated titration ratios of CAR-modified T cells, mock-transduced T cells and naive CD3^+^ T cells at approximate E:T ratios were added for another 24 h. MTT values were evaluated using absorbance at 570 nm on an ELISA reader (BioTek, Winooski, VT, USA). The viability of cells was calculated as the percentage of MTT reduction. All experiments were performed at least in triplicate on three separate occasions. Data are presented as means ± SDs.

### Life Cell Monitoring Assay

The xCELLigence system was used to monitor cell survival according to the instructions of the supplier (Roche Diagnostics, Mannheim, Germany, and ACEA Bioscience, San Diego, CA, USA). Cells were grown for 24 h, with impedance measured every hour prior to treatments following manufacturer’s instructions.^54^ Cell impedance is represented by CI = (Zi − Z0) ohms/15 ohms, where Zi is the impedance at an individual time point and Z0 is background resistance. A normalized CI was determined as the CI at a certain time point divided by the CI at the normalization time point.

### Cytokine Release Assays

Mouse serum samples were obtained at serial time points by using the retro-orbital plexus bled. Cytokine profiles were determined using the Human Cytokine/Chemokine Magnetic Bead Panel protocol from the Milliplex Map Kit (Cat. No. HCYTOMAG-60K; Billerica, MA, USA). In brief, cytokine/chemokine assay plates were washed, sealed, and mixed on an orbital plate shaker for 10 min at room temperature. After the addition of samples or controls, samples were incubated overnight at 4°C on an orbital shaker with fluorescently labeled capture antibody-coated beads, which were used for the simultaneous quantification of the following 41 human cytokines and chemokines in serum samples according to manufacturer’s recommendations: EGF, eotaxin, granulocyte colony-stimulating factor (G-CSF), granulocyte-macrophage colony-stimulating factor (GM-CSF), IFN-alpha 2, IFN-γ, IL-10, IL-12P70, IL-13, IL-15, IL-17A, IL-1RA, IL-1α, IL-1β, IL-2, IL-3, IL-4, IL-5, IL-6, IL-7, IL-8, IP-10, MCP-1, macrophage inflammatory protein (MIP)-1α, MIP-1β, regulated upon activation normal T cell expressed and secreted (RANTES), TNF-α, TNF-β, vascular endothelial growth factor (VEGF), FGF-2, transforming growth factor (TGF)-α, farnesyltransferase inhibitor (FIT)-3L, fractalkine, GRO, MCP-3, MDC, PDGF-AA, PDGF-AB/BB, sCD40L, and IL-9. Plates were run on the Luminex MagPix machine, and data were collected using the Luminex xPONENT software (v.4.2). The cytokine/chemokine level was analyzed using the Milliplex Analyst software (v.5.1).

### Xenograft and Orthotopic Models of Human Pancreatic Carcinoma

Six- to eight-week-old nude (BALB/cAnN.Cg-Foxn1nu/CrlNarl) mice were maintained in a specific pathogen-free facility according to National Institutes of Health guidelines for animal care and the guidelines of Taipei Medical University, Taipei, Taiwan. All animal experiments were performed according to protocols reviewed and approved by the Institutional Animal Care and Use Committee/Institutional Animal Care and Use Program (IACUC/IACUP; protocol no.: LAC-2015-0251, December 22, 2015). For established s.c. CFPAC1 models, mice were inoculated s.c. with 1 × 10^7^ and/or 1.5 × 10^7^ CFPAC1 cells on the left flank on day 0. Moreover, for establishing orthotopic CFPAC1 models, orthotopic inoculation of 7.5 × 10^6^ CFPAC1 cells was undertaken into the caudal pancreas of mice on day 0. Mice were then randomly assigned into five treatment groups (n = 6 mice/group): (1) Ctrl (1× PBS) only without T cells; (2) non-transduced CD3 T cells in 1× PBS; (3) genetically modified mock T cells in 1× PBS (mock); (4) genetically modified PD1ACR-T cells in 1× PBS; and (5) genetically modified PDL1CAR-T cells (1 × 10^6^ cells/mouse) through an i.v. injection. Tumor dimensions were measured using calipers for s.c. models, and tumor volumes were calculated using the following formula: V = 1/2 (length × width^2^). Orthotopic tumor growth was determined once a week using an IVIS (Perkin Elmer, Boston, MA, USA) with an intraperitoneal (i.p.) injection of 200 μL D-luciferin (30 mg/mL). At the endpoint dissection, xenografts were measured and fixed with formalin, embedded in paraffin, and sections were cut and processed for immunohistochemical (IHC) staining.

### Immunohistochemistry and Scoring of Immunoreactivity

Solid tumors were excised from sacrificed tumor xenograft mice under sterile conditions at indicated times after treatment. For immunohistochemistry, serial tissue sections (4 μm thick) were prepared from formalin-fixed tumor samples and mounted on glass slides. After rehydration, sample sections were stained with monoclonal antibodies against human CD3, PD-1, IgG1, CD44, and Ki67. After 10 min of 3,3′-diaminobenzidine incubation, tumors were counterstained with hematoxylin. Images were acquired using an Olympus microscope (BX50; Tokyo, Japan). For semiquantitative analysis of the immunoreactivity of markers, H-score was used, as previously described.^55^ In brief, more than 10 fields were counted in each case, and the H-score was subsequently generated by adding the percentages of strongly stained (3×), moderately stained (2×), and weakly stained (1×) cells, giving a possible range of 0−300.

### Statistical Analysis

Data are expressed as the means ± standard deviations of three independent experiments. A two-tailed Student’s t test was used for intergroup comparisons. Comparisons between groups were determined with a one-way analysis of variance. Differences were considered significant at p < 0.05 by using SPSS v.13.0 software.

## Author Contributions

C.L.L. designed and performed the research, analyzed the data, and wrote the paper. C.-Y.Y. and M.H.F. performed the research and analyzed the data. C.H.M. designed the project and wrote the paper. Y.J.L. and R.-H.Y. conducted the experiments.

## Conflicts of Interest

The authors declare no competing interests.

## Supplemental Information

Document S1. Figures S1–S3

Document S2. Article plus Supplemental Information
