# Supplementary material for: Engineering Chimeric Antigen Receptor T Cells against Immune Checkpoint Inhibitors PD-1/PD-L1 for Treating Pancreatic Cancer
Source: Mol Ther Oncolytics. 2020 May 26;17:571–85. doi: 10.1016/j.omto.2020.05.009 (PMC7321819; doi:10.1016/j.omto.2020.05.009)
Supplement: Document S1. Figures S1–S3 [file mmc1.pdf]

OMTO, Volume *17*

Supplemental Information

Engineering Chimeric Antigen Receptor T Cells against Immune Checkpoint Inhibitors PD-1/PD-L1 for Treating Pancreatic Cancer

Ching-Yao Yang, Ming Huei Fan, Carol H. Miao, Yi Jen Liao, Ray-Hwang Yuan, and Chao Lien Liu

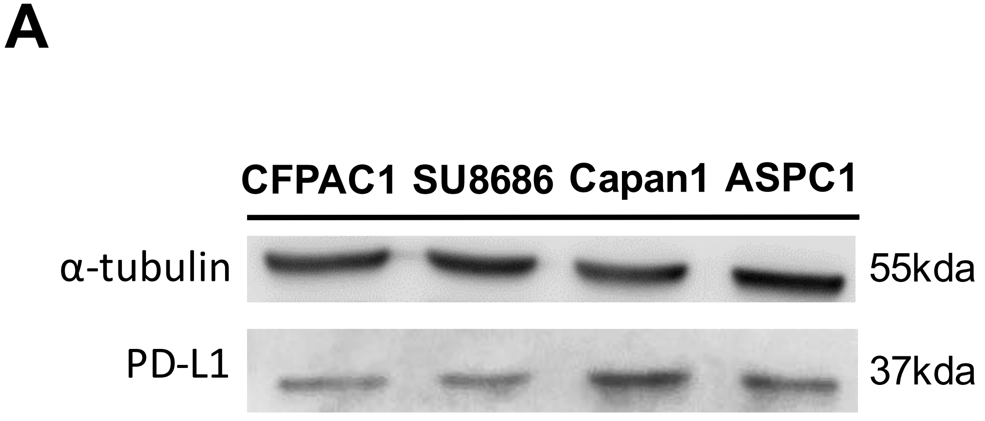

Ratio (PD-L1/ α-tubulin)	CFPAC1	SU8686	Capan1	Aspc1
	0.261	0.238	0.605	0.317

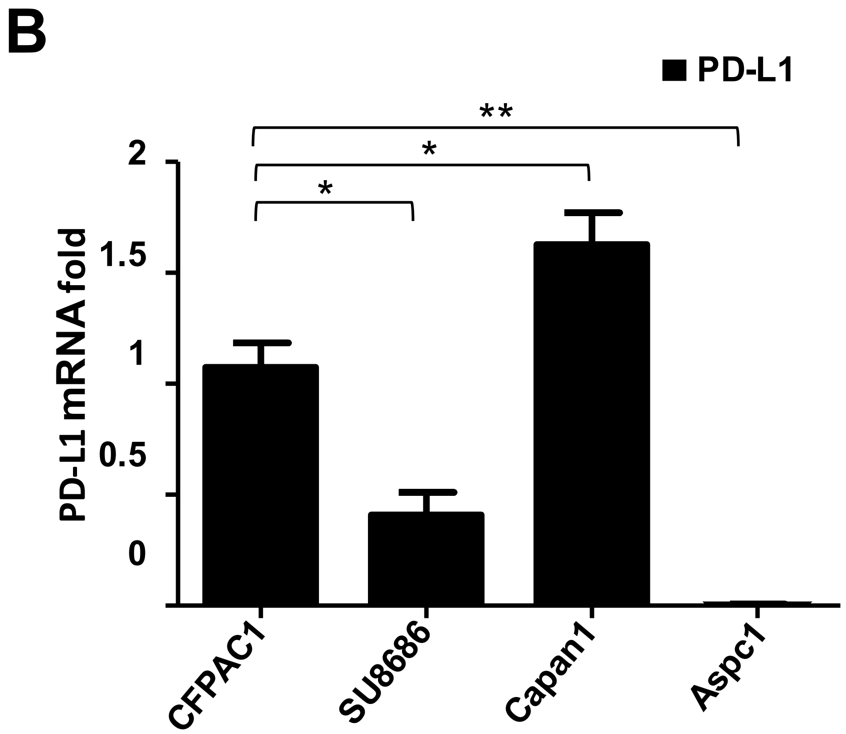

Figure S1. PD-L1 expressions in human pancreatic cancer cell lines. **A**, a western blot analysis showed PD-L1 protein expressions among CFPAC1, SU8686, Capan1 and ASPC1 comparison. Alpha-tubulin served as a loading control. Data shown are the PD-L1/α-tubulin ratios. **B**, PD-L1 mRNA levels among the same four pancreatic cancer cell lines comparison using a real-time PCR analysis. Alpha-tubulin was used as a loading control (**p* < 0.05; ***p* < 0.01). Data are presented at least of three independent experiments.

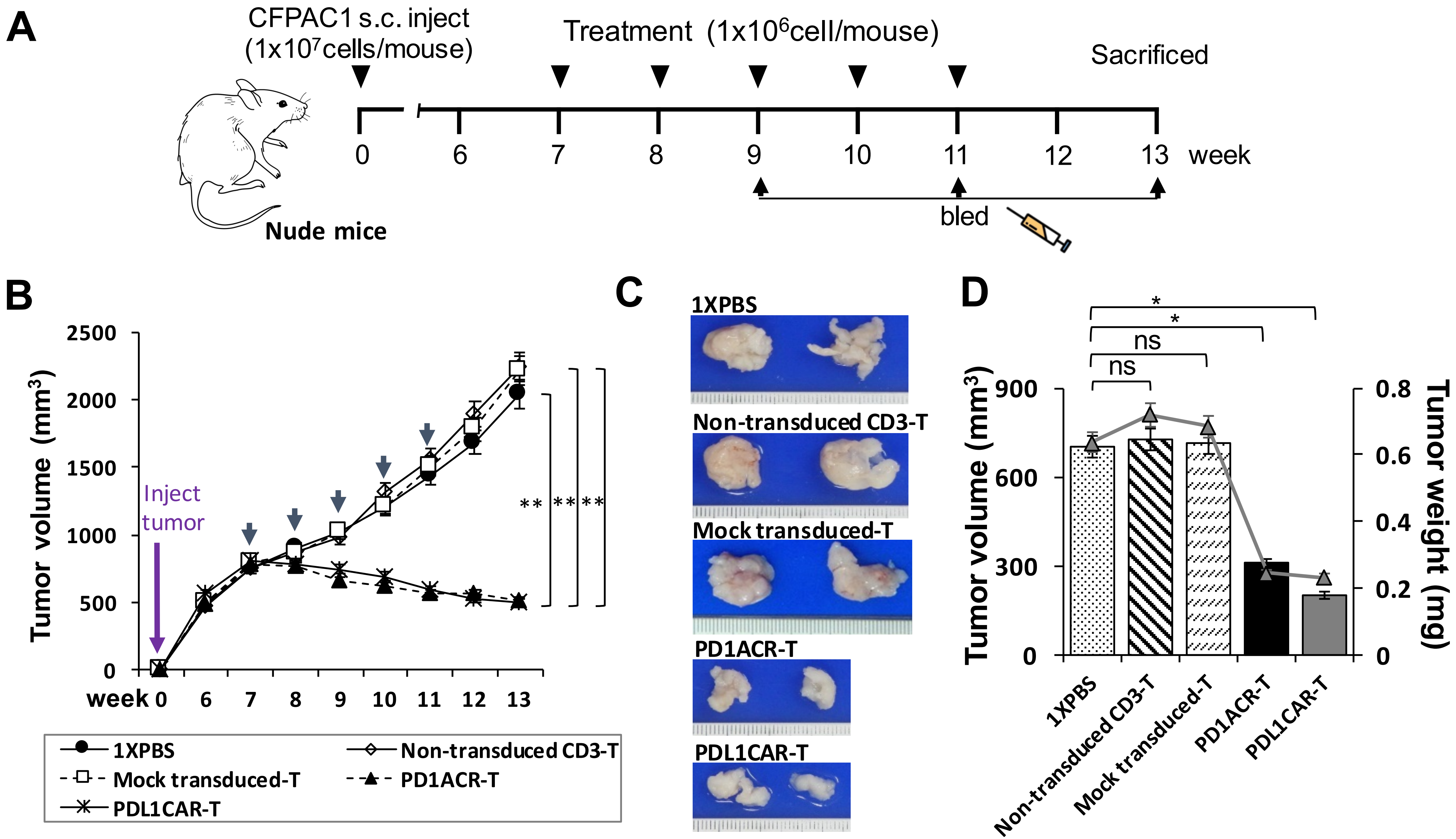

Figure S2. *In vivo* antitumor activities of PD1ACR-T and PDL1CAR-T cells on established subcutaneous pancreatic tumor xenograft. **A**, a detailed treatment schedule of in vivo study was shown. **B**, growth curve of CFPAC1 xenografts treated with the indicated T cells or 1xPBS. At the endpoint (week 13), the residual tumors treated with PD1ACR-T and PDL1CAR-T cells were significantly smaller than those in the control groups, respectively (n = 4 per group; $^{**}p < 0.01$). **C** and **D**, the endpoint dissection of treated mice. Tumor masses (C), the mean tumor volume (mm3) and mean tumor weight (mg) (D) from each treated mouse group (n = 4 per group; $^{*}p < 0.05$, respectively).

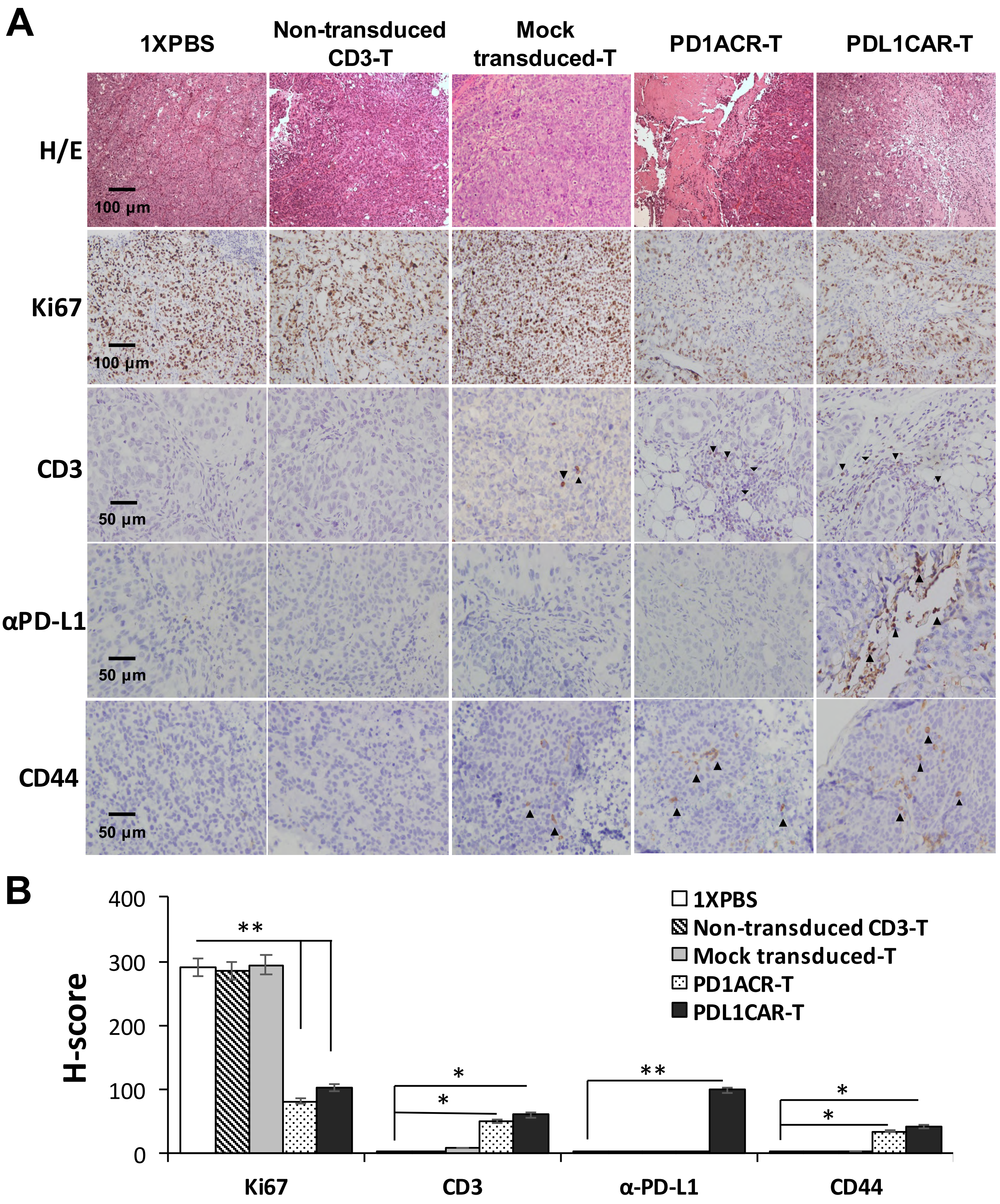

Figure S3. Adoptively transferred PD1ACR-T and PDL1CAR-T cells could be located in CFPAC1 tumors. Tumors were collected from mice bearing CFPAC1 subcutaneous xenografts treated with PD1ACR-T cells, PDL1CAR-T cells, mock-transduced control, non-transduced CD3 T cells, or 1xPBS. **A,** Formalin-fixed, paraffin-embedded tumor sections were consecutively cut and stained for H/E, Ki67, CD3, anti-PDL1 IgG, and CD44 expressions (brown dots and/or black arrow). The image were taken with the microscope (BX50, Olympus, Tokyo, Japan) and camera (DP22) under x 400 or x 200 magnifications. Individual scale bars were shown. **B**, The average H-score for each marker and the comparison between groups are shown (*$p < 0.05$; **$p < 0.01$).
